# Analysis of the Performance of Recycled Insulation Concrete and Optimal Mix Ratio Design Based on Orthogonal Testing

**DOI:** 10.3390/ma16165688

**Published:** 2023-08-18

**Authors:** Jun-Xi Deng, Xiao Li, Xiao-Juan Li, Tai-Bing Wei

**Affiliations:** 1 College of Transportation and Civil Engineering, Fujian Agriculture and Forestry University, Fuzhou 350108, China; 3211342007@fafu.edu.cn (J.-X.D.); 3201342019@fafu.edu.cn (X.L.); xiaojuanli@fafu.edu.cn (X.-J.L.); 2 Department of Civil Engineering and Architecture, Wuyi University, Wuyishan 354300, China

**Keywords:** construction solid waste, straw fiber, glazed hollow beads, insulation performance, orthogonal test, total efficiency coefficient method

## Abstract

Construction and agricultural waste recycling have gained more and more attention recently as renewable resources. Straw and construction waste, both of which are widespread in northern Fujian, were investigated in this research. The orthogonal test was used to investigate the effects of recycled aggregate, straw, and glazed hollow beads on the mechanical and thermal properties of recycled insulation concrete. The influence of different factors on the macroscopic characteristics of recycled insulation concrete was examined using scanning electron microscopy (SEM). The optimal mix proportion for recycled insulation concrete that satisfies mechanical performance standards and provides superior insulation performance was then determined using the total efficacy coefficient method. According to the research findings, the heat conductivity of recycled insulation concrete decreases as its dried density decreases. A 100% recycled coarse aggregate replacement rate, 1% straw content, and 10% glazed hollow beads replacement rate are the optimal mix ratios for recycled insulation concrete. With a compressive strength of 20.98 MPa, a splitting tensile strength of 2.01 MPa, a thermal conductivity of 0.3776 W/(m·K), and a dry density of 1778.66 kg/m^3^, recycled insulation concrete has the optimal mix ratio. Recycled insulation concrete is a novel form of eco-friendly, energy-saving concrete that aims to achieve low-carbon energy savings and sustainable development by combining resource recycling with building energy savings to realize the recycling of solid waste resources, which has significant environmental, social, and economic benefits and broad market application potential.

## 1. Introduction

China’s environmental pollution and energy shortages have become increasingly conspicuous due to the country’s burgeoning economy and construction industry. On the one hand, the frequent remodeling, tearing down, and new construction of buildings have produced a large volume of construction solid waste [1,2,3]. With the current supply of sand, stone, and other natural resources, which is relatively tight, the use of construction solid waste to prepare recycled concrete can achieve partial recycling of construction solid waste [4,5,6]. According to statistics, every 10,000 square meters of construction in China will generate 500~600 t of construction waste materials, but the recycling rate is only 5%. The use of construction solid waste for resource utilization is the basic direction and inevitable requirement of the development of the construction industry [7,8,9]. Other than that, buildings need a lot of energy to operate and maintain. Making maximum use of waste resources and creating innovative energy-saving materials is especially significant in the current energy crisis. One of the key ways to achieve energy savings in buildings is to implement insulation measures that will lower the energy consumption produced by structures [10,11]. The building envelope, as the most important part of buildings’ operation and energy consumption, will constitute a breakthrough to solve the technical problem of buildings’ thermal insulation and energy savings [12].

Numerous studies on the mechanical properties of recycled concrete have been conducted in recent years, but not many on its thermal properties [13,14,15,16]. During the investigation of the factors affecting the thermal conductivity of recycled concrete, Xiao [1] discovered that the physical properties of recycled aggregates have some influence on the thermal conductivity of recycled concrete. The thermal conductivity of concrete is reduced when the recycled aggregates have greater water absorption and lesser density (i.e., when the porosity is greater). Using orthogonal testing, Zhu et al. [17] examined the effects of water consumption per unit volume, the water–cement ratio, and the replacement rates of recycled coarse aggregate and recycled fine aggregate on the density and thermal conductivity of recycled concrete. The replacement rate of recycled coarse aggregate was found to have the greatest impact on the thermal conductivity of recycled concrete. The second consideration was the replacement rate for recycled fine aggregate. The ratio of water to cement and the water consumption per unit of volume had minimal effects. The varied physical properties of recycled aggregates from various sources can also result in varying thermal conductivity. Laneyrie et al. [18] compared the effects of recycled coarse aggregates generated in a factory versus in a laboratory on the thermal conductivity of concrete. The thermal conductivity of the two varieties of recycled concrete was found to be identical at ambient temperature and with the same replacement rate of recycled coarse aggregate. However, the thermal conductivity of the concrete mixed with laboratory-prepared recycled coarse aggregate was 13% greater as compared to the latter when the recycled concrete was heated to 300 °C. The use of recycled aggregates to make recycled concrete is an important direction for developing environmentally friendly thermal insulation building materials. This method of recycling concrete not only resolves the issue of environmental degradation brought on by a lack of naturally occurring aggregates and significant amounts of sand and gravel mining, it also has good thermal insulation performance, which contributes to a reduction in the emission of greenhouse gases, the consumption of natural resources, and the cost of production [8,19,20].

Plant fiber has numerous advantages over conventional fiber, including low cost, green environmental protection, and convenient materials. Plant-fiber concrete is a novel form of eco-friendly concrete [21]. The main method used in China to deal with waste straw is burning; however, this method wastes renewable resources like straw, and it will cause pollution to worsen and the quality of the air to degrade annually if there are too many incinerations [22,23]. Li [24,25] added various proportions and varieties of straw fibers to the same concrete mixture and discovered that the compressive strength, splitting tensile strength, and flexural strength of the concrete decreased despite the addition of straw fibers, but its toughness increased. Liu [26] investigated the effects of filamentous and rod-like straw fiber mixed with fly ash on the mechanical properties of concrete. It has been shown that when the amount of rice straw fiber and fly ash increases, the compressive strength, splitting tensile strength, and flexural strength of concrete decrease. However, rice straw fiber may significantly improve the brittle failure mode of concrete. In the investigation of the thermal insulation capabilities of straw concrete, Song [27] discovered that as the percentage of fly ash in the mixture increased, the thermal insulation capabilities of the test blocks increased, but their compressive strength decreased. Chen et al. [28] investigated the working characteristics, mechanical characteristics, and thermal insulating qualities of concrete when straw and fly ash were combined. They found that adding straw to concrete can decrease its strength, apparent density, slump, and thermal conductivity. Rape straw concrete is better than wheat straw and corn concrete in terms of workability, strength, and thermal insulation efficiency.

Glazed hollow bead insulation concrete is ordinary concrete to which glazed hollow beads (which are lightweight insulation materials) and admixtures have been added. With the typical physical and mechanical capabilities of concrete, as well as thermal insulation properties, this new type of concrete is an eco-friendly building material [29,30,31]. Zhang [32] successfully developed glazed hollow bead insulation concrete with strength grade C35 and a thermal conductivity of 0.206 W/(m·K). It has also been proven that it is feasible to use glazed hollow beads as a thermal insulation material in concrete. In terms of research on the mix ratio of glazed hollow bead insulation concrete, Zhang [33] developed an orthogonal experiment on the mix ratio of self-insulated recycled concrete based on the total efficiency coefficient method and investigated the impacts of various factors on the compressive strength and thermal conductivity of recycled insulation concrete. Through design optimization, recycled insulating concrete with a 28 d compressive strength of 19.0 MPa and a thermal conductivity of 0.429 W/(m·K) was developed. Wang et al. [34] mixed glazed hollow beads into recycled concrete at a volume ratio of 1:1 and discovered that the thermal conductivity of recycled insulation concrete could reach 0.322 W/(m·K) at its lowest, which is considerably lower than the thermal conductivity of ordinary concrete, and that its mechanical properties and durability were excellent.

This study aimed to develop a new type of building material for the building envelope that not only meets the mechanical requirements of the building envelope but also provides superior thermal insulation. By using an orthogonal test, the impacts of recycled aggregate, straw, and hollow glazed beads on the mechanical and thermal characteristics of recycled insulation concrete were investigated. Based on the test findings, the mechanical and thermal characteristics of recycled insulation concrete were assessed, and the ideal mix ratio was established using the total efficiency coefficient technique. Compared to conventional concrete, recycled insulation concrete can recycle construction waste and agricultural waste, thereby reducing resource waste and achieving resource recycling in accordance with the principles of sustainable green development [35,36,37]. To hasten the industrialization of building structures, save energy, and lessen environmental contamination, it is essential to investigate innovative wall materials.

## 2. Materials and Testing Method

### 2.1. Material

This research employed P·O 42.5 R grade cement with an apparent density of 3100 kg/m^3^. Table 1 lists the chemical composition of the cement.

**Figure 1:**
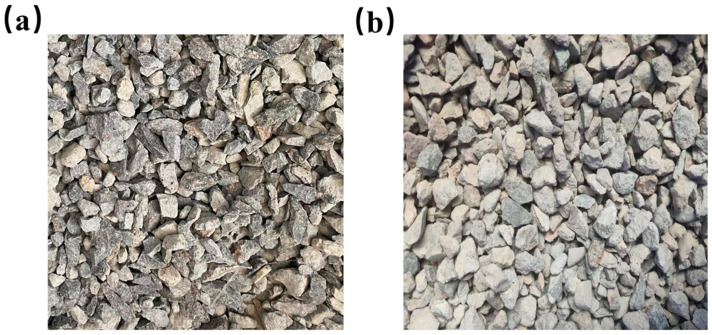
Aggregates: (**a**) natural coarse aggregate of 5~16 mm particle size; (**b**) recycled coarse aggregate of 5~16 mm particle size.

**Figure 2:**
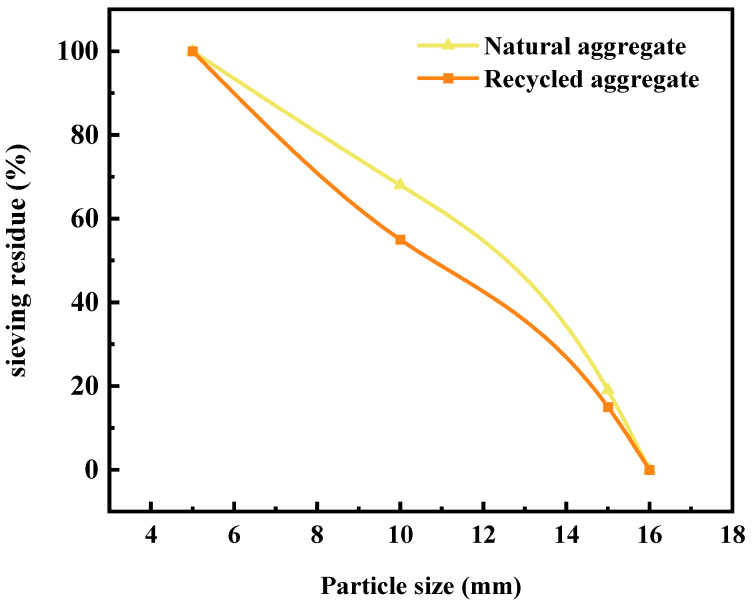
Sieving curve of coarse aggregates.

As illustrated in Figure 1a, the natural coarse aggregate used in the experiment was sourced from the raw gravel around Wuyi Mountain, which was cleaned and screened by the gravel field before being further screened by the laboratory screening equipment to extract coarse aggregate. As shown in Figure 1b, the recycled coarse aggregate was derived from the waste concrete of pavement improvement projects, which was initially crushed in the crushing plant and then further screened by the laboratory screening machine. Table 2 displays the physical properties of the coarse aggregates. The aggregate sieving curve is shown in Figure 2.

**Figure 3:**
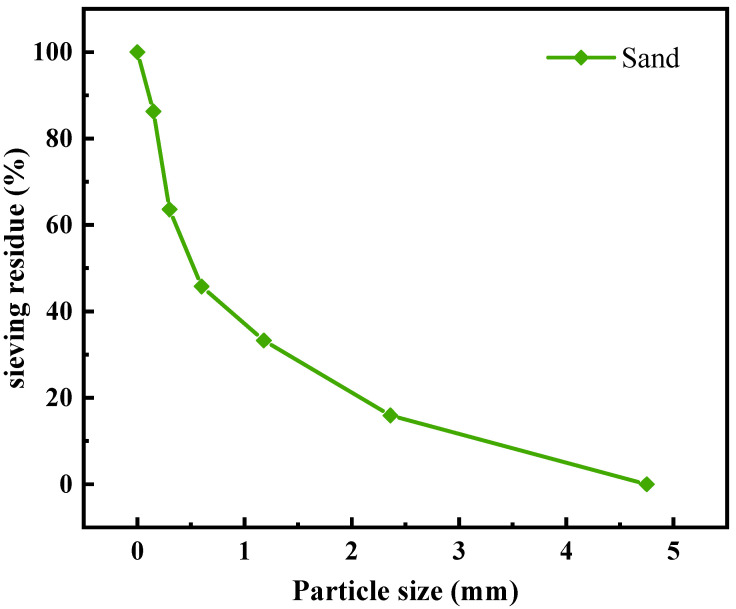
Sieving curve of fine aggregates.

The sand for this experiment was natural river sand supplied by Wuyi Mountain sand and gravel manufacturers, with a particle size of 0~5 mm, an apparent density of 2610 kg/m^3^, and a water absorption rate of 1.51%, as determined by sieving. Figure 3 depicts the sieving curves for natural sand.

**Figure 4:**
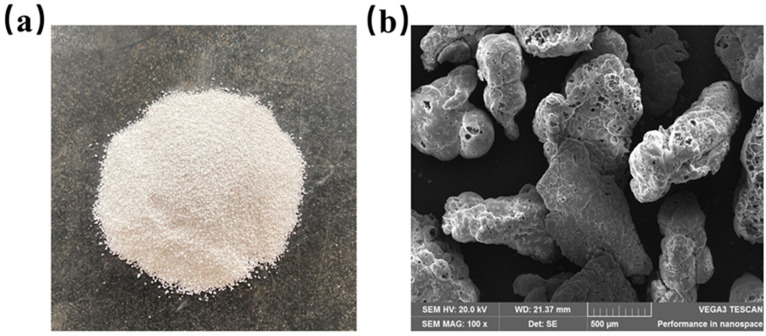
Glazed hollow beads: (**a**) the apparent morphology of glazed hollow beads with a particle size of 0.5~1.5 mm; (**b**) the microscopic morphology of glazed hollow beads magnified 100×.

Glazed hollow beads from Henan Xinyang Chenxin New Material Co., Ltd., Xinyang, China were chosen for this test. On the exterior of the glazed hollow bead material are irregular spherical particles, while the interior has a porous cavity structure. The surface is glazed, smooth, level, and closed. It is an inorganic and lightweight insulation material with excellent functionality and sustainability. Figure 4a depicts the apparent morphology of the glazed hollow beads, whereas Figure 4b depicts their microscopic morphology. Table 3 lists the physical properties of the glazed hollow beads.

**Figure 5:**
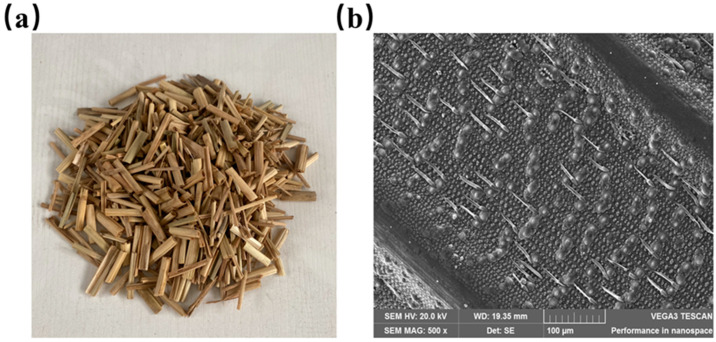
Straw: (**a**) the apparent morphology of straw with a length of 1~3 cm; (**b**) microscopic morphology of straw magnified 500×.

The straw was composed of rice straw, which is commonly in northern Fujian, and the rice straw was manually sheared to eliminate the fiber portion. The length of the straw was 1~3 cm, and its width was 2~3 mm. The straw fibers needed to be washed with tap water 2–3 times, drained, dried, and stored in a dry location to prevent moisture. Figure 5a depicts the apparent morphology of the straw, while Figure 5b depicts the microscopic morphology of the straw. Table 4 shows the physical property parameters of the straw.

Jiangsu Subote New Materials Co., Ltd., Nanjing, China manufactures polycarboxylate superplasticizer with a water reduction rate of 25~30%.

### 2.2. Basic Mix Ratio Design

As there are no relevant regulations for the mix ratio design, the recycled insulation concrete studied in this experiment is a new type of thermal insulation material that is primarily deemed to be used in the building envelope, based on its intended application. Recycled insulation concrete contains light aggregate which are glazed hollow beads in place of a portion of sand that is found in sand lightweight concrete. The preliminary foundation mix design was carried out in accordance with the “Ordinary Concrete Mix Design Regulations [38]” and “Light Aggregate Concrete Technical Regulations [39]”. According to Specification GBT1490-94, “Light Aggregate Concrete Technical Regulations”, the acceptable range of concrete strength for envelope structures primarily used for both load-bearing and thermal insulation is LC5~LC15. Due to the fact that the addition of straw, recycled coarse aggregate, and glazed hollow beads will significantly reduce the strength of the concrete, the design of the foundation mix ratio should account for sufficient strength loss. As a result, C30 was allocated as the reference concrete’s strength level. In Table 5, the tested basic mix ratio is displayed.

### 2.3. Breakage Rate Test of Glazed Hollow Beads

**Figure 6:**
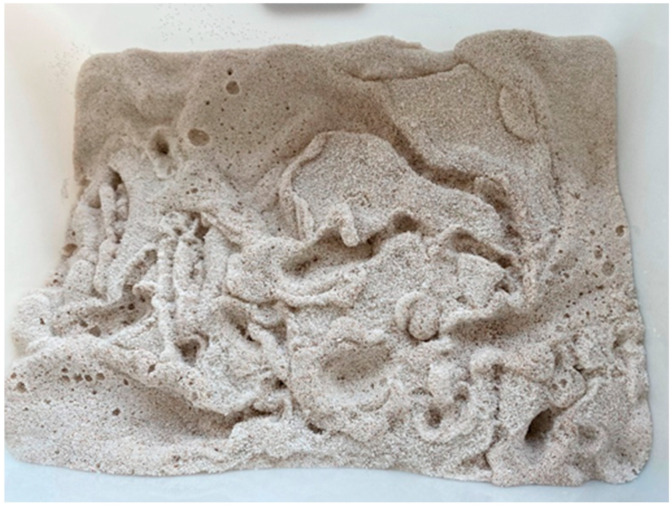
Pre-wetting treatment of glazed hollow beads.

The water absorption rate of the glazed hollow beads in 24 h is 230%, according to Table 2. In order to prevent the water absorption rate of glazed hollow beads from being excessively high during the concrete blending process, moisture from the cement slurry is absorbed. Figure 6 illustrates that the glazed hollow beads are pre-wetted for 2~3 h prior to mixing the concrete. The glazed hollow beads are in a saturated state of water absorption during the mixing of the concrete, and the concrete’s water-to-cement ratio is kept constant. In the process of blending the concrete, in addition to the glazed hollow beads, there are admixtures such as aggregate and sand, the strength of which exceeds that of glazed hollow beads. Therefore, the glazed hollow beads that have been saturated with water will be compressed and rubbed, causing some of them to break during the concrete mixing process. The water used to pre-wet the glazed hollow beads will precipitate along with their damage, and the precipitated water will cause the water–cement ratio of the mixed concrete to be too high, which will lead to segregation. Therefore, the actual water consumption of concrete mixed with glazed hollow beads needs to subtract the moisture precipitated by the damage to the glazed hollow beads [40,41].

**Figure 7:**
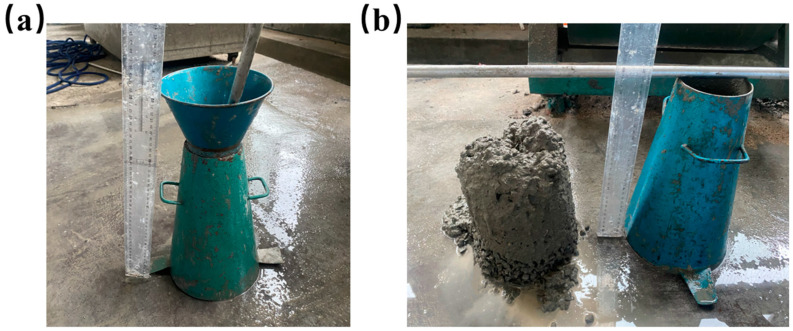
Slump: (**a**) slump test equipment; (**b**) slump test diagram.

The breakage rate of the glazed hollow beads was measured based on the basic mix ratio of the concrete. Four experimental groups were conducted, in which glazed hollow beads replaced 0%, 5%, 10%, and 15% of the sand mass in the basic mix ratio of the concrete. Table 6 displays the mix proportions of the four test groups. In all four test groups, the same water reducer was used, and the slump of the foundation concrete mix group (145 ± 10 mm) was used as the measurement standard. Figure 7 depicts the diagram of the slump test.

The quantity of water released from the breakage of glazed hollow beads and the breakage rate of glass beads were calculated as shown in Equations (1) and (2):

(1) $$W_{b}=W_{n}-W_{m}$$

(2) $$B=\frac{W_{b}}{W_{p}}$$

where $W_{b}$ is the amount of water released when the glazed hollow beads are broken during the concrete mixing process, $W_{n}$ is the amount of water required for the concrete foundation mix to reach (145 ± 10) mm slump, $W_{m}$ is the amount of water added in the mixing process of glazed hollow bead concrete when the slump is (145 ± 10) mm, $W_{p}$ is the pre-wetting water consumption of the glazed hollow beads, and B is the rate of breakage for the glazed hollow beads.

**Figure 8:**
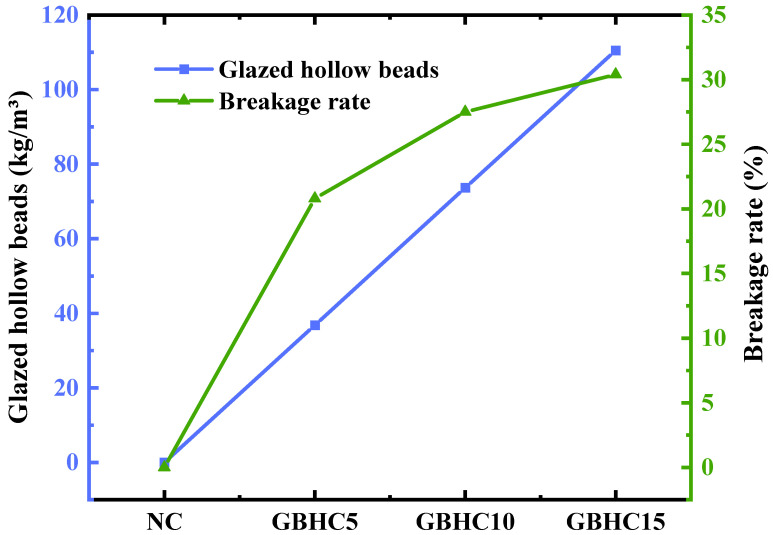
Variation in breakage rate with the amount of glazed hollow beads.

According to Table 7, the breakage rate for the 5% to 15% replacement rates of glazed hollow beads varies between 20.8% and 30.0%. Figure 8 demonstrates that the rate of breakage increases as the amount of glazed hollow beads increases. This is due to the fact that the cement slurry might cover the surface of the glazed hollow beads when their content is low. The glazed hollow beads can provide cushioning when squeezed during the mixing of the concrete. The breakage rate steadily rises as a result of the glazed hollow beads being rubbed and squeezed by other admixtures as the number of glazed hollow beads is gradually increased and the cement slurry is unable to completely envelop the glazed hollow beads.

### 2.4. Orthogonal Test Design

When compared to natural coarse aggregate, recycled coarse aggregate absorbs water almost three times as well. The additional water consumption of recycled coarse aggregate was calculated in order to lessen the effect of the high water absorption of recycled coarse aggregate on the effective water–cement ratio. [42]. Equation (3) was used to compute the increased amount of water used by the recycled aggregate, while the actual water consumption of recycled insulation concrete was calculated using Equation (4):

(3) $$W_{r}=W_{ra}-W_{na}\times Q_{ra}$$

(4) $$W_{a}=W_{c}+W_{r}-W_{b}$$

where $W_{r}$ denotes the extra water that the recycled coarse aggregate uses, $W_{ra}$ is the water absorption rate of the recycled aggregate, $W_{na}$ is the water absorption rate of the natural aggregate, $Q_{ra}$ is the quality of the recycled aggregate, $W_{a}$ is the actual water consumption, and $W_{c}$ is the net water consumption.

This test utilizes the orthogonal test method, with compressive strength, splitting tensile strength, thermal conductivity, and dry density serving as test indicators; the orthogonal table design is depicted in Table 8 [43]. Three factors were chosen: the replacement rate of recycled aggregate, the straw content, and the replacement rate of glazed hollow beads. As shown in Table 9, four levels were chosen for each factor, and the orthogonal table L16 (4^3^) was utilized. The content of glazed hollow beads is the mass percentage of replaced sand, the content of recycled coarse aggregate is the mass percentage of recycled coarse aggregate replacing natural aggregate, and the content of straw is the mass percentage of cement.

### 2.5. Test Preparation and Performance Testing

According to Standard GB/T 50081-2002 for Mechanical Properties of Ordinary Concrete [44], the mechanical experiments were carried out. The size of the test block was 100 mm × 100 mm × 100 mm. The test block’s compressive strength and splitting tensile strength were determined after the block had been cured for 28 days in a standard curing environment (temperature of 20 ± 2 °C and humidity of 95%).

The thermal performance of the recycled insulation concrete was determined using the GB/T 10294-2008 standard [45]; 300 mm (length) × 300 mm (width) × 30 mm (thickness) were the dimensions of the test block. The dry density of the test block of recycled insulation concrete, which was oven-baked to a consistent weight by the dryer, was calculated after 28 d of curing in the standard curing chamber. The thermal conductivity of the test block was then measured using a JTRG-III thermal conductivity instrument. The thermal conductivity tester’s cold plate temperature and hot plate temperature were adjusted to 15 °C and 35 °C, respectively, once the test block had reached room temperature. After the test results stabilized, the data were collected.

Scanning electron microscopy (SEM) is mainly employed for observing the surface morphology of specimens via secondary electron signal imaging or backscattered electron imaging [46]. After the mechanical test of the concrete specimen, dried sample particles from the central portion of the fractured specimen were selected for microstructural analysis. A conductive adhesive was used to adhere the sample to the special sample table of the SEM apparatus. After spraying the sample with gold, SEM was carried out. The appearance, pore morphology, and state of failure of samples were determined using SEM.

## 3. Results and Analysis

### 3.1. Results

Table 10 and Table 11 display the orthogonal test results.

### 3.2. Range Analysis

**Figure 9:**
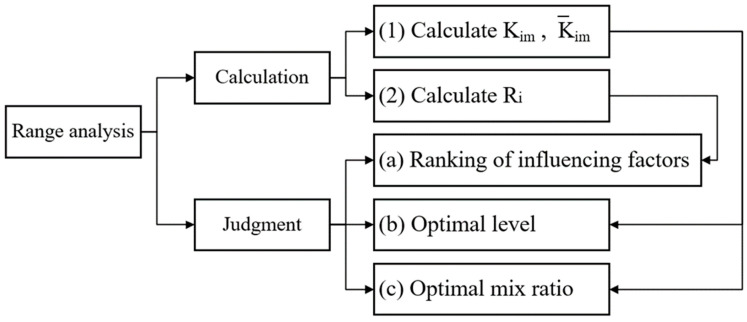
Range analysis diagram.

Range analysis includes two steps: calculation and judgment. The diagram of range analysis is shown in Figure 9. K_im_ is the sum of test indicators corresponding to the m level of factor i, where  i  = A, B, C and m  = 1, 2, 3, 4. Factors and levels are shown in Table 8. $\bar{K}$_im_ indicates the average value of K_im_. The optimal level and optimal mix ratio of factor i can be judged by the size of $\bar{K}$_im_. R_i_ represents the degree to which a change in the level of factor  i affects the test index, R*_i_* = $\bar{K}$_max_ $-\;\bar{K}$_min_. The greater the R_i_, the greater the factor’s influence on the test indicator and, consequently, the greater its importance [47].

#### 3.2.1. Range Analysis of Mechanical Properties

As shown in Table 12, the range analysis method was used to analyze the mechanical property test results.

According to Table 12, the factors that influence the compressive strength are in the following order: glazed hollow beads > straw > recycled aggregate. The optimal compressive strength scheme is A_1_B_1_C_1_ according to the $\bar{K}$*_im_* values. The sequence of factors affecting splitting tensile strength is as follows: glazed hollow beads > straw > recycled aggregate. The optimal scheme of splitting tensile strength is A_1_B_1_C_1_. In conclusion, glazed hollow beads are the primary factors influencing the mechanical properties of recycled concrete.

#### 3.2.2. Range Analysis of Insulation Properties

According to Table 13, the range value R_i_ is as follows: 0.5699 (glazed hollow beads) > 0.3013 (recycled aggregate) > 0.1277 (straw). The following is the ranking of the factors that influence thermal conductivity: glazed hollow beads > straw > recycled aggregate. A_4_B_4_C_4_ has the greatest thermal insulation performance, according to the $\bar{K}$*_im_*.

### 3.3. Variance Analysis

Despite the fact that range analysis is straightforward, it has limitations and cannot estimate the size of the experimental error. Therefore, analysis of variance was carried out on the results of the orthogonal experiment to determine whether the change in the test results was due to the change in factor levels or to the error [48].

#### 3.3.1. Variance Analysis of Mechanical Properties

The variance analysis of mechanical properties is illustrated in Table 14.

According to Table 14, $F_{A}=5.170,\;F_{B}=5.497,\;$ and$\;F_{c}=36.528$. The order of each factor’s impact on compressive strength is as follows: glazed hollow beads (C) > straw (B) > recycled aggregate (A), which is consistent with the range analysis’s findings. In accordance with the F values of the variables influencing the splitting tensile strength, $F_{A}=1.953,\;F_{B}=1.098,\;$ and $F_{c}=23.592$. In accordance with the findings of the range analysis, the order of influence of each factor on splitting tensile strength is as follows: glazed hollow beads (C) > straw (A) > recycled aggregate (B).

#### 3.3.2. Variance Analysis of Insulation Properties

The variance analysis of insulation performance is depicted in Table 15.

According to Table 15, the F values calculated by each factor are $F_{A}=15.197,\;F_{B}=2.787$, and $F_{c}=56.660$, and the influence of each factor on the thermal conductivity is as follows: glazed hollow beads (C) > recycled aggregate (A) > straw (B). The effect of glazed hollow beads on the thermal conductivity is significantly greater than that of recycled aggregate. Consistent with the results of the range analysis, straw has some impact on thermal conductivity.

### 3.4. Relationship between Thermal Conductivity and Dry Density 

**Figure 10:**
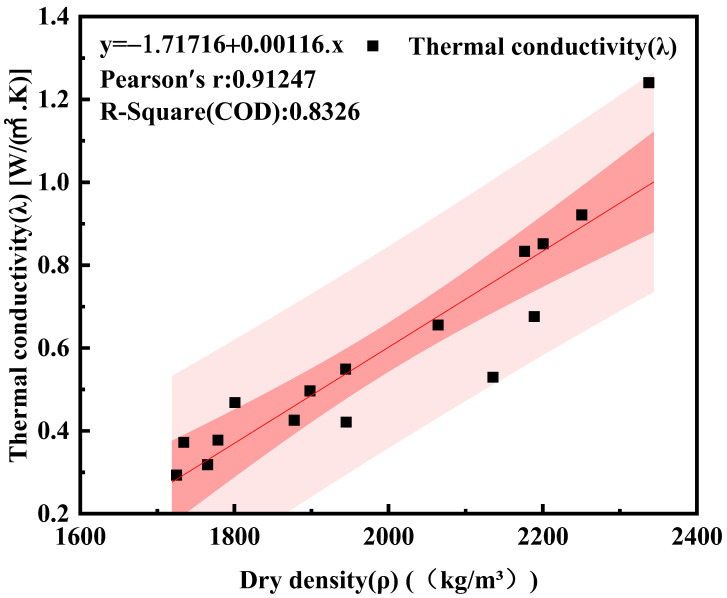
The relationship between thermal conductivity and dry density. The darker pink is the confidence band, and the lighter pink is the prediction band.

Figure 10 illustrates the association between thermal conductivity and dry density. As shown in the graph, the dry density affects the thermal insulation effectiveness of recycled insulation concrete: as the dry density increases, so does the thermal conductivity. This is due to the fact that as the dry density of recycled insulation concrete increases, the porosity decreases and the compactness rises, resulting in an increase in thermal conductivity. By using least squares regression, it is feasible to obtain Equation (5) of thermal conductivity and dry density, and the correlation coefficient R^2^ = 0.8326.

(5) y=0.00116x−1.71716

### 3.5. Optimal Mix Ratio Design

In order to find a balance between the four test indicators selected for this experiment and determine the mix ratio of recycled insulation concrete that provides excellent thermal insulation performance while meeting the requirements for mechanical properties, the total efficiency coefficient method was utilized to analyze the four test indicators [49]. As a result, the test had *j* groups and n indicator kinds. The normalized value of the jth set of test data that corresponds to indicator i is $d_{ji}$, and the greatest test value that corresponds to indicator i is $C_{max}$. The lowest test value is $C_{min}$.

In the test, benefit indicators such as compressive strength and splitting tensile strength were used, with a higher result indicating a greater benefit. Formula (6) was used to normalize the mechanical characteristics of the concrete, such as splitting tensile strength and compressive strength. Thermal conductivity and dry density are forms of cost indicators for which lesser values are preferable. The thermal conductivity and dry density of concrete, which were normalized according to Equation (7), represent the insulation properties of the concrete [50].

Benefit indicator normalization calculation (the greater, the better):

(6) $$d_{ji}=\frac{C_{ji}}{C_{max}}$$

Cost indicator normalization calculation (the smaller, the better):

(7) $$d_{ji}=\frac{C_{min}}{C_{ji}}$$

The total efficacy coefficient of the group j test was determined using the single efficiency coefficient of the evaluation indicator:

(8) $$D_{j}=\sqrt[{n}]{d_{j1}d_{j2}\ldots d_{j,m-1}d_{jm}}$$

The highest total efficiency coefficient was found in group 14, as indicated in Table 16, where $D_{14}=0.692$. As a result, the 14th group’s mix ratio, which had a recycled aggregate replacement rate of 100%, a straw content of 1%, and a glazed hollow beads replacement rate of 10%, was chosen as the optimal mixture ratio. The optimal mix ratio of recycled insulation concrete has compressive strength, splitting tensile strength, thermal conductivity, and dry density of 20.98 MPa, 2.01 MPa, 0.3776 W/(m·K), and 1778.66 kg/m^3^, respectively. The strength of recycled insulation concrete fulfills the GBT1490-94 “Technical Specifications for Light Aggregate Concrete” specification, which states that “the reasonable range of concrete strength for both load-bearing and thermal insulation envelope structures is LC5~LC15”. Under the condition of meeting the standard’s mechanical property requirements, the thermal conductivity of recycled insulation concrete is 0.3776 W/(m·K), which is 69.56% less than that of ordinary concrete, which is 1.2405 W/(m·K). Under the condition that the mix ratio satisfies the code’s requirements for mechanical properties, it has superior thermal insulation performance.

## 4. Discussion

### 4.1. Effects of the Single Factors on Mechanical Properties

**Figure 11:**
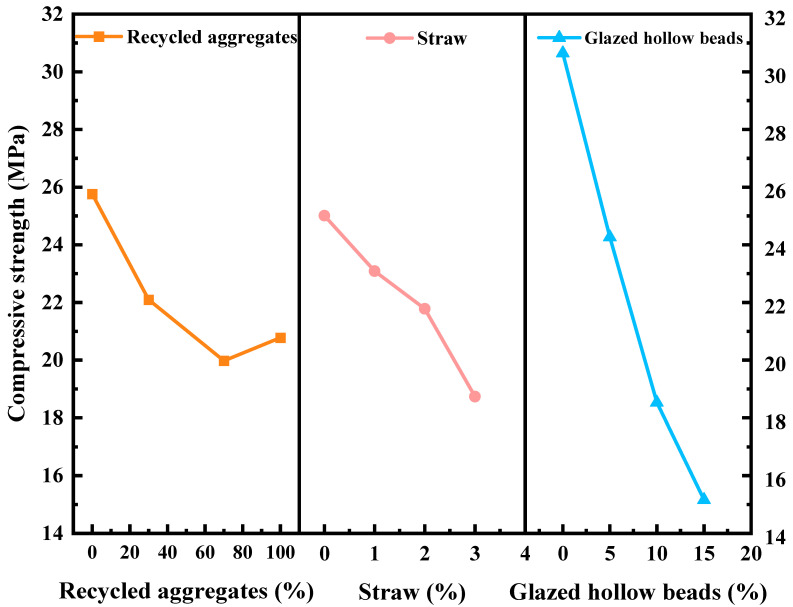
Influence of test factors on compressive strength.

**Figure 12:**
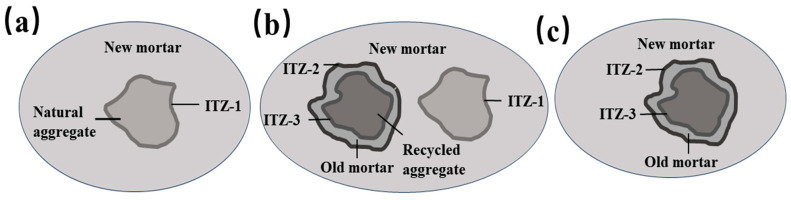
ITZs: (**a**) ITZ of ordinary concrete; (**b**) ITZ of recycled concrete; (**c**) ITZ of fully recycled concrete.

**Figure 13:**
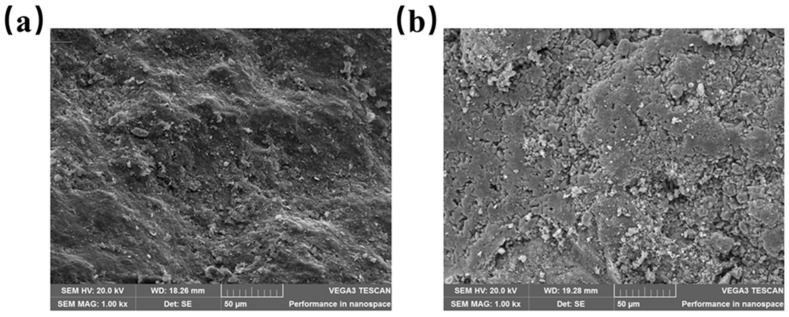
Aggregates’ microstructure: (**a**) natural aggregate magnified 1.00 kx; (**b**) recycled aggregate magnified 1.00 kx.

**Figure 14:**
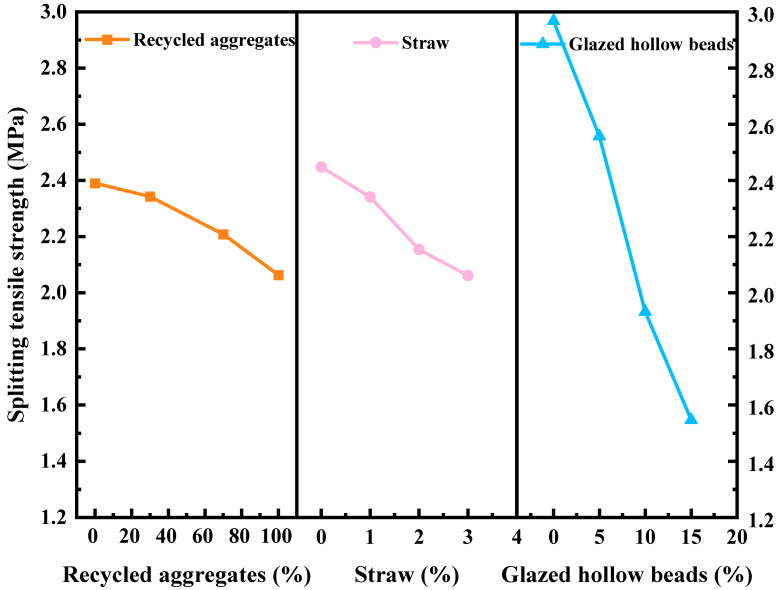
Influence of test factors on the splitting tensile strength.

According to Figure 11, the compressive strength of recycled insulation concrete decreases by 23.04% when the proportion of recycled aggregate being replaced rises to 70% from 0%. However, the increase is 3.85% when the substitute rate of recycled aggregate is raised from 70% to 100%. In general, compressive strength decreases as the substitution rate of recycled aggregates rises. As shown in Figure 12a, when the recycled aggregate substitution rate is 0%, there is a single interface transition zone (ITZ), ITZ-1 (the interfacial transition zone between the natural aggregate and the new mortar). Figure 12b illustrates the substitution rate of recycled aggregate when it is between 30% and 70%. The concrete has three interfacial transition zones, namely, ITZ-1, ITZ-2 (the interfacial transition zone between new mortar and old mortar), and ITZ-3 (the interfacial transition zone between recycled aggregate and old cement mortar). As depicted in Figure 12c, ITZ-2 and ITZ-3 are the interfacial transition zones of concrete when 100% recycled aggregate is utilized. The interfacial transition zone is the area where cracks occur and develop in the concrete, as well as being the weakest area of the concrete [51,52]. Figure 13 illustrates the SEM morphology of two coarse aggregate categories. As a result of the fact that recycled aggregate is produced by pulverizing construction waste, the figure shows that recycled aggregate contains more dust and leftover cement mortar on its surface than does natural aggregate. The ITZ between the old mortar and the new mortar has high porosity and a loose structure, resulting in an unstable connection between the new mortar and the recycled aggregate. Therefore, the mechanical properties of recycled insulation concrete degrade with increasing recycled coarse aggregate content [53,54]. According to Figure 14, the splitting tensile strength decreases progressively as the recycled aggregate replacement rate increases. The splitting section typically occurs at a vulnerable interface, such as the zone of transition between cement mortar and aggregate. The development of splitting tensile cracks accelerates as the replacement rate of recycled aggregate rises gradually [55].

**Figure 15:**
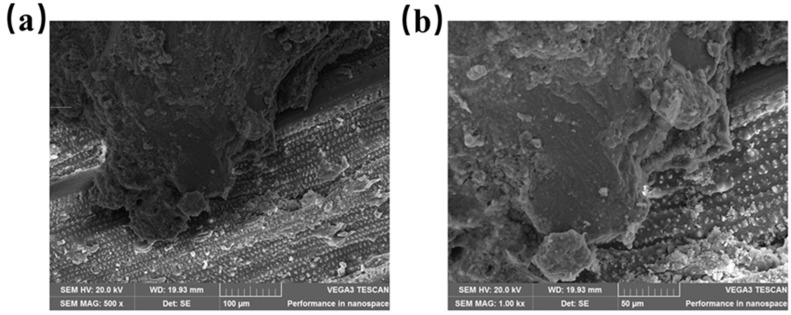
ITZ between straw and cement mortar: (**a**) magnified 500×; (**b**) magnified 1.00 kx.

As shown in Figure 11 and Figure 14, the mechanical properties of recycled insulation concrete decrease as the straw content rises. The compressive strength and splitting tensile strength of recycled insulation concrete decreased by 25.11% and 12.33%, respectively, as the straw content increased from 0% to 3%. Since the primary components of straw fiber are hemicellulose, lignin, and other substances, these substances readily dissolve a portion of the sugar in an alkaline environment and hinder the growth of Ca (OH)_2_ crystals, which causes the cement slurry to solidify and harden more slowly and to exhibit the “slow coagulation” phenomenon, so that the mechanical properties are diminished [56]. Under the SEM, Figure 15 depicts the ITZ between straw and cement mortar in recycled insulation concrete. The figure demonstrates that the addition of straw will increase the internal porosity of concrete, thereby decreasing its mechanical properties [3,48].

As shown in Figure 10 and Figure 13, the mechanical properties of recycled insulation concrete decrease as the percentage of glazed hollow beads increases. The compressive strength and splitting tensile strength of recycled insulation concrete decreased by 50.54% and 47.8%, respectively, when the replacement rate of glazed hollow beads was increased from 0% to 15%. In this investigation, some sand in the concrete was replaced with glazed hollow beads. The glazed hollow beads are light porous cavity structures, and their strength is relatively low compared to that of sand [29,41]. In addition, as the amount of glazed hollow beads increases, it becomes easier to form channels between glazed hollow bead particles, and more and more closed pores of glazed hollow beads are distributed in the concrete, resulting in a reduction in its compressive strength and splitting tensile strength [57].

### 4.2. Effects of the Single Factors on Thermal Conductivity

**Figure 16:**
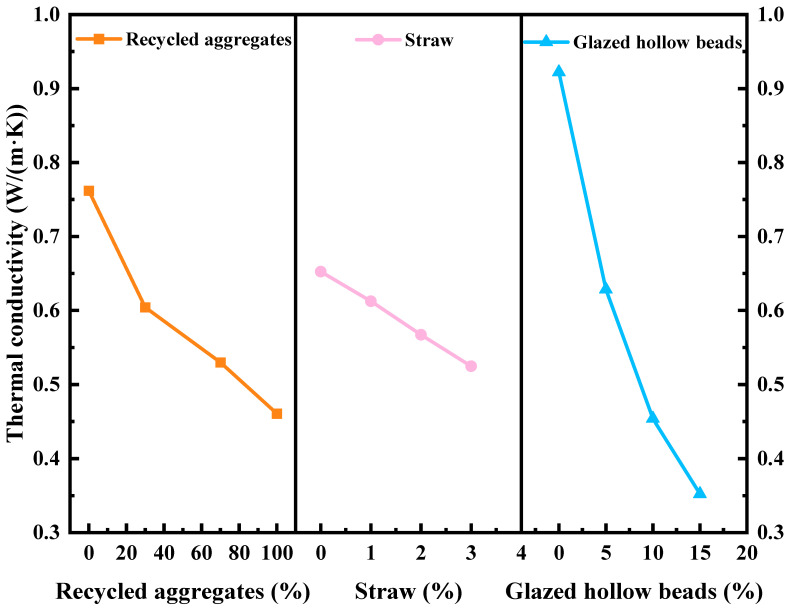
Influence of test factors on thermal conductivity.

Figure 16 demonstrates that when the replacement rate of recycled aggregate increases from 0% to 100%, the thermal conductivity of recycled insulation concrete decreases by 39.54%, indicating that the thermal insulation performance continues to improve. Xiao [1] found that the thermal conductivity of concrete can be reduced by 29% when 100% recycled coarse aggregate is used to replace natural coarse aggregate. Recycled aggregate has more porosity and greater water absorption than natural aggregate, as it is made from the crushing of building debris and contains a significant proportion of old cement mortar that has been adsorbed on the surface [58]. Consequently, when the recycled aggregate substitution rate rises, the thermal conductivity falls.

As the proportion of straw in recycled insulation concrete increased from 0% to 3%, its thermal conductivity decreased by 19.57%. As seen in the microtopography of the straw in Figure 5b, honeycomb pores exist within the fiber, and the primary components of the straw, such as lignin, cellulose, etc., are poor thermal conductors, resulting in excellent thermal insulation performance [59]. In addition, the addition of straw will increase the internal pores of concrete, and the greater the number of closed interior pores, the lower the thermal conductivity and the better the thermal insulation performance [56].

**Figure 17:**
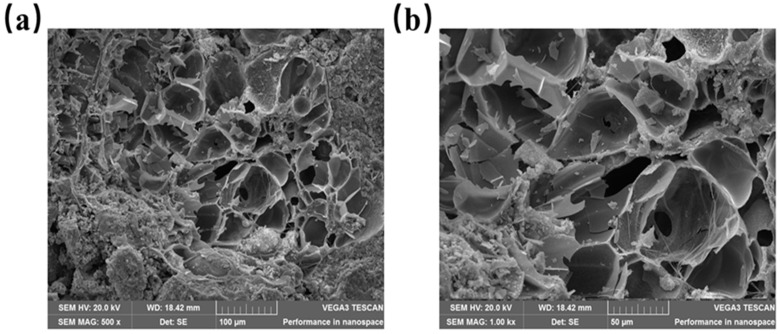
Microtopography of glazed hollow beads in concrete: (**a**) magnified by 500×; (**b**) magnified by 1.00 kx.

The thermal conductivity of the recycled insulation concrete decreased by 61.80% as the replacement rate of glazed hollow beads increased from 0% to 15%. As depicted in Figure 17, the microtopography of the glazed hollow beads within the recycled insulation concrete reveals a porous cavity structure. The internal holes of the glazed hollow beads in the recycled insulation concrete are filled with air, and the thermal conductivity of air is 0.024 W/(m·K). The glazed hollow beads medium can inhibit and slow down the transfer of heat flow when the concrete conducts heat. Therefore, the greater the addition of glazed hollow beads, the more evidently the thermal conductivity of the recycled insulation concrete decreases; that is, the better its thermal insulation performance [60].

## 5. Conclusions

The preliminary investigation of this novel type of recycled insulation concrete yielded the following findings and prospects:

- The following describes the degree of influence for mechanical properties: glazed hollow beads trump recycled aggregate and straw. In terms of thermal conductivity, glazed hollow beads have a greater impact than recycled aggregate and straw.
- The optimal mix ratio of recycled insulation concrete was found in the 14th orthogonal test group. The recycled aggregate replacement rate in this group was 100%, the straw content was 1%, and the glazed hollow beads replacement rate was 10%. The compressive strength of recycled insulation concrete with this optimal mix ratio is 20.98 MPa, the splitting tensile strength is 2.01 MPa, the thermal conductivity is 0.3776 W/(m·K), and the dry density is 1778.66 kg/m^3^.
- The equation for the relationship between thermal conductivity and dry density is y=0.00116x−1.71716. As the dry density decreases, the thermal conductivity decreases.
- The macro performance and internal microstructure were simply combined in this investigation. In the future, macroscale, mesoscale, and microscale can be combined to study the internal heat transfer mechanism of insulation concrete more thoroughly.
- This study only takes into account the mechanical properties of recycled insulation concrete; future studies may take into account the durability of recycled insulation concrete in order to apply it to various building structures and increase the rate at which solid waste resources are utilized.

## Figures and Tables

**Table 1:** Chemical components of the cement.

Components	SiO_2_	CaO	Al_2_O_3_	Fe_2_O_3_	SO_3_	MgO	Loss
Content (%)	20.30	60.39	5.10	4.30	2.24	2.57	2.1

**Table 2:** The physical characteristics of the coarse aggregates.

Type	Particle Size (mm)	Packing Density (kg/m^3^)	Apparent Density (kg/m^3^)	Crushing Indicator (%)	Water Absorption (%)
Natural coarse aggregate	5~16	1442	2660	7.5	1.1
Recycled coarse aggregate	5~16	1320	2380	14.5	3.4

**Table 3:** Physical properties of the glazed hollow beads.

Particle Size (mm)	Stacking Density (kg/m^3^)	Cylinder Compression Strength (Kpa)	Thermal Conductivity (W/(m·K))	24 h Water Absorption (%)	Surface Vitrified Closed Porosity (%)	Volume Flotation Rate (%)
0.5~1.5	114	191	0.041	230	95	91

**Table 4:** Physical performance parameters of the straw.

Dry Bulk Density (kg.m^3^)	Wet Bulk Density (kg/m^3^)	Loose Bulk Density (kg/m^3^)	Compacting Bulk Density (kg/m^3^)	Porosity (%)	Mass of Water Absorption (%)	Volume of Water Absorption (%)	Thermal Conductivity (W/(m·K))
191 ± 17	108 ± 6	164 ± 13	164 ± 13	82 ± 5	353 ± 16	207 ± 23	0.09

**Table 5:** Basic mix ratio of recycled insulation concrete.

Materials	Water (kg/m^3^)	Cement (kg/m^3^)	Fine Aggregate (kg/m^3^)	Natural Coarse Aggregate (kg/m^3^)	Water Reducer (kg/m^3^)
content	200	420	736.75	1016.7	4.2

**Table 6:** Mix ratios of glazed hollow beads’ breakage rate tests.

Materials	Normal Concrete (NC)	5% Glazed Hollow Beads Replacement Rate (GHBC5)	10% Glazed Hollow Beads Replacement Rate (GHBC10)	15% Glazed Hollow Beads Replacement Rate (GHBC15)
Cement (kg/m^3^)	420	420	420	420
Natural coarse aggregate (kg/m^3^)	1016.7	1016.7	1016.7	1016.7
Sand (kg/m^3^)	736.75	699.91	663.075	626.24
Glazed hollow beads (kg/m^3^)	0	36.84	73.675	110.51
Water reducer (kg/m^3^)	4.2	4.2	4.2	4.2

**Table 7:** Water release and breakage rate of glazed hollow beads.

Concrete Parameter	NC	GHBC5	GHBC10	GHBC15
Slump (mm)	145	136	150	142
Pre-wetting water consumption (kg/m^3^)	0	84.73	169.45	254.17
Stirring water consumption (kg/m^3^)	200	182.36	153.40	122.73
Breakage release water volume $W_{b}$ (kg/m^3^)	0	17.64	46.60	77.27
Breakage rate (B)	0	20.82	27.50	30.40

**Table 8:** Factors and levels of the orthogonal test design.

	Level	1	2	3	4
Factor					
(A) Recycled coarse aggregate (%)		0	30	70	100
(B) Straw (%)		0	1	2	3
(C) Glazed hollow beads (%)		0	5	10	15

**Table 9:** Orthogonal test table of recycled insulation concrete.

Number	Cement (kg/m^3^)	Natural Coarse Aggregate (kg/m^3^)	Sand (kg/m^3^)	Recycled Coarse Aggregate (kg/m^3^)	Straw (kg/m^3^)	Glazed Hollow Beads (kg/m^3^)	Actual Water Consumption (kg/m^3^)	Water Reducer (kg/m^3^)
1	420	1016.7	736.75	0	0	0	200	4.2
2	420	1016.7	699.91	0	4.2	36.84	182.37	4.2
3	420	1016.7	663.07	0	8.4	73.68	153.4	4.2
4	420	1016.7	626.24	0	12.6	110.51	122.73	4.2
5	420	711.7	699.91	305	0	36.84	189.39	4.2
6	420	711.7	736.75	305	4.2	0	207.02	4.2
7	420	711.7	626.24	305	8.4	110.51	129.75	4.2
8	420	711.7	663.07	305	12.6	73.68	160.42	4.2
9	420	305	663.07	711.7	0	73.68	169.77	4.2
10	420	305	626.24	711.7	4.2	110.51	139.10	4.2
11	420	305	736.75	711.7	8.4	0	216.27	4.2
12	420	305	699.91	711.7	12.6	36.84	198.75	4.2
13	420	0	626.24	1016.7	0	110.51	146.11	4.2
14	420	0	663.07	1016.7	4.2	73.68	176.78	4.2
15	420	0	699.91	1016.7	8.4	36.84	205.76	4.2
16	420	0	736.75	1016.7	12.6	0	223.38	4.2

**Table 10:** Orthogonal test results of mechanical properties.

Number	Recycled Aggregates (A)/%	Straw (B)/%	Glazed Hollow Beads (C)/%	Compressive Strength (MPa)	Splitting Tensile Strength (MPa)
1	0	0	0	38.73	3.58
2	0	1	5	27.87	2.84
3	0	2	10	20.53	1.90
4	0	3	15	15.90	1.25
5	30	0	5	26.59	2.69
6	30	1	0	31.32	2.98
7	30	2	15	15.93	1.87
8	30	3	10	14.54	1.83
9	70	0	10	18.08	1.98
10	70	1	15	12.17	1.53
11	70	2	0	28.86	2.73
12	70	3	5	20.81	2.58
13	100	0	15	16.65	1.54
14	100	1	10	20.98	2.01
15	100	2	5	21.80	2.12
16	100	3	0	23.68	2.58

**Table 11:** Orthogonal test results of thermal performance.

Number	Recycled Aggregates (A)/%	Straw (B)/%	Glazed Hollow Beads (C)/%	Thermal Conductivity (W/(m·K))	Dry Density (kg/m^3^)
1	0	0	0	1.2405	2337.85
2	0	1	5	0.8332	2176.64
3	0	2	10	0.5486	1944.29
4	0	3	15	0.4256	1877.59
5	30	0	5	0.6552	2064.37
6	30	1	0	0.9213	2250.75
7	30	2	15	0.3723	1734.34
8	30	3	10	0.4683	1800.81
9	70	0	10	0.4209	1945.10
10	70	1	15	0.3182	1765.41
11	70	2	0	0.8512	2200.59
12	70	3	5	0.5293	2135.47
13	100	0	15	0.2931	1725.13
14	100	1	10	0.3776	1778.66
15	100	2	5	0.4964	1898.14
16	100	3	0	0.6756	2188.95

**Table 12:** Range analysis of mechanical properties.

Indicators	K	Recycled Aggregate (A)	Straw (B)	Glazed Hollow Beads (C)
Compressive strength	K _i1_	103.04	100.04	122.60
	K _i2_	88.37	92.35	97.07
	K _i3_	79.92	87.12	74.13
	K _i4_	83.11	74.94	60.65
	$\bar{K}$ _i1_	25.76	25.01	30.65
	$\bar{K}$ _i2_	22.09	23.09	24.27
	$\bar{K}$ _i3_	19.98	21.78	18.53
	$\bar{K}$ _i4_	20.78	18.73	15.16
	R _i_	5.78	6.28	15.49
	Optimization	A_1_B_1_C_1_		
Splitting tensile strength	K _i1_	9.56	9.79	11.87
	K _i2_	9.37	9.36	10.23
	K _i3_	8.83	8.62	7.73
	K _i4_	8.25	8.24	6.19
	$\bar{K}$ _i1_	2.39	2.45	2.97
	$\bar{K}$ _i2_	2.34	2.34	2.56
	$\bar{K}$ _i3_	2.21	2.15	1.93
	$\bar{K}$ _i4_	2.06	2.06	1.55
	R _i_	0.33	0.39	1.42
	Optimization	A_1_B_1_C_1_		

**Table 13:** Range analysis of thermal conductivity.

Indicator	K	Recycled Aggregate (A)	Straw (B)	Glazed Hollow Beads (C)
Thermal conductivity	K _i1_	3.0479	2.6097	3.6886
	K _i2_	2.4171	2.4503	2.5141
	K _i3_	2.1196	2.2685	1.8154
	K _i4_	1.8427	2.0988	1.4092
	$\bar{K}$ *_i_*_1_	0.7620	0.6524	0.9222
	$\bar{K}$ *_i_*_2_	0.6043	0.6126	0.6285
	$\bar{K}$ *_i_*_3_	0.5299	0.5671	0.4539
	$\bar{K}$ *_i_*_4_	0.4607	0.5247	0.3523
	R *_i_*	0.3013	0.1277	0.5699
	Optimization	A_4_B_4_C_4_		

**Table 14:** Variance analysis of mechanical properties.

Indicators	Factors	SS	df	MS	F	F_CV_	Sig
Compressive strength	Recycled aggregate (A)	78.482	3	26.161	5.170	F_0.01_ (3,6) = 9.78	*
	Straw (B)	83.453	3	27.818	5.497	F_0.05_ (3,6) = 4.76	*
	Glazed hollow beads (C)	554.514	3	184.838	36.528	F_0.1_ (3,6) = 3.29	**
	e	30.361	6	5.060		F_0.2_ (3,6) = 2.1	
	e ^△^	746.810	15				
Splitting tensile strength	Recycled aggregate (A)	0.260	3	0.087	1.165	F_0.01_ (3,6) = 9.78	Δ
	Straw(B)	0.369	3	0.123	1.652	F_0.05_ (3,6) = 4.76	Δ
	Glazed hollow beads (C)	4.824	3	1.608	21.595	F_0.1_ (3,6) = 3.29	**
	e	0.447	6	0.074		F_0.2_ (3,6) = 2.1	
	e ^Δ^	5.900	15				

**Note:** SS: total deviation from the mean sum of squares; df: degree of freedom; MS: mean square; F: statistic; F_CV_: the critical value of F; Sig: significance. “e”: error; “e^Δ^”: total error; “Δ”: some influence; “*”: significance; “**”: high significance.

**Table 15:** Variance analysis of insulation performance.

Indicator	Factors	SS	df	MS	F	F_CV_	Sig
Thermal conductivity	Recycled aggregate (A)	0.200	3	0.067	15.197	F_0.01_ (3,6) = 9.78	**
	Straw (B)	0.037	3	0.012	2.787	F_0.05_ (3,6) = 4.76	Δ
	Glazed hollow beads (C)	0.747	3	0.249	56.660	F_0.1_ (3,6) = 3.29	**
	*e*	0.026	6	0.004		F_0.2_ (3,6) = 2.1	
	*e* ^Δ^	1.011	15				

**Note:** SS: total deviation from the mean sum of squares; df: degree of freedom; MS: mean square; F: statistic; F_CV_: the critical value of F; Sig: significance. “e”: error; “e^Δ^”: total error; “Δ”: some influence; “**”: high significance.

**Table 16:** Normalized treatment and indicator test results.

Number	Compressive Strength (MPa)	Splitting Tensile Strength (MPa)	Thermal Conductivity (W/(m·K))	Dry Density (Kg/m^3^)	Total Efficiency Coefficient
1	38.73 (1.000)	3.58 (1.000)	1.2405 (0.236)	2337.85 (0.738)	0.646
2	27.87 (0.720)	2.84 (0.793)	0.8332 (0.352)	2176.64 (0.793)	0.632
3	20.53 (0.530)	1.90 (0.530)	0.5486 (0.534)	1944.29 (0.887)	0.604
4	15.90 (0.411)	1.25 (0.349)	0.4256 (0.689)	1877.59 (0.919)	0.549
5	26.59 (0.686)	2.69 (0.753)	0.6552 (0.447)	2064.37 (0.836)	0.663
6	31.32 (0.809)	2.98 (0.833)	0.9213 (0.318)	2250.75 (0.766)	0.637
7	15.93 (0.411)	1.87 (0.521)	0.3723 (0.787)	1734.34 (0.995)	0.640
8	14.54 (0.375)	1.83 (0.512)	0.4683 (0.626)	1800.81 (0.958)	0.583
9	18.08 (0.467)	1.98 (0.554)	0.4209 (0.696)	1945.10 (0.887)	0.632
10	12.17 (0.314)	1.53 (0.429)	0.3182 (0.921)	1765.41 (0.977)	0.590
11	28.86 (0.745)	2.73 (0.764)	0.8512 (0.344)	2200.59 (0.784)	0.626
12	20.81 (0.537)	2.58 (0.722)	0.5393 (0.554)	2135.47 (0.808)	0.645
13	16.65 (0.430)	1.54 (0.430)	0.2931 (1.000)	1725.13 (1.000)	0.656
14	20.98 (0.542)	2.01 (0.563)	0.3776 (0.776)	1778.66 (0.970)	0.692
15	21.80 (0.563)	2.12 (0.592)	0.4964 (0.590)	1898.14 (0.909)	0.650
16	23.68 (0.612)	2.58 (0.721)	0.6756 (0.434)	2188.95 (0.788)	0.623

## Data Availability

Not applicable.
